# *Trpm2* Ablation Accelerates Protein Aggregation by Impaired ADPR and Autophagic Clearance in the Brain

**DOI:** 10.1007/s12035-018-1309-0

**Published:** 2018-09-13

**Authors:** Yongwoo Jang, Byeongjun Lee, Hyungsup Kim, Seungmoon Jung, Sung Hoon Lee, So-Young Lee, Ji Hyun Jeon, In-Beom Kim, Seo-Ho Lee, Byung-Ju Kim, Uh-Hyun Kim, Yunjong Lee, Sung Min Kim, Daejong Jeon, Uhtaek Oh

**Affiliations:** 1 College of Pharmacy, Seoul National University, Seoul, 02862 South Korea; 2 Department of Psychiatry and Program in Neuroscience, McLean Hospital, Harvard Medical School, Belmont, MA 02478 USA; 3 Department of Biomedical Engineering, Hanyang University, Seoul, 04763 South Korea; 4 Sensory Research Center, Brain Science Institute, Korea Institute of Science and Technology, 5 Hwarang-ro 14-gil, Seongbuk-gu, Seoul, 02792 South Korea; 5 Department of Bio and Brain Engineering, Korea Advanced Institute of Science and Technology, Daejeon, 305-701 South Korea; 6 College of Pharmacy, Chung-ang University, Seoul, 06974 South Korea; 7 Department of Anatomy, College of Medicine, Catholic University of Korea, Seoul, 137-701 South Korea; 8 Department of Biochemistry, College of Medicine, Chonbuk National University, Jeonju, South Korea; 9 Department of Molecular Cell Biology, Sungkyunkwan University School of Medicine, Samsung Biomedical Research Institute, Suwon, 446–746 South Korea; 10 Department of Active Aging Industry, Hanyang University, Seoul, 04763 South Korea; 11 Department of Neurology, Comprehensive Epilepsy Center, Biomedical Research Institute, Seoul National University Hospital, Seoul, 110-744 South Korea

**Keywords:** ADPR, AMP, Autophagy, Protein aggregation, TRPM2

## Abstract

TRPM2 a cation channel is also known to work as an enzyme that hydrolyzes highly reactive, neurotoxic ADP-ribose (ADPR). Although ADPR is hydrolyzed by NUT9 pyrophosphatase in major organs, the enzyme is defective in the brain. The present study questions the role of TRPM2 in the catabolism of ADPR in the brain. Genetic ablation of *Trpm2* results in the disruption of ADPR catabolism that leads to the accumulation of ADPR and reduction in AMP. *Trpm2*^−/−^ mice elicit the reduction in autophagosome formation in the hippocampus. *Trpm2*^−/−^ mice also show aggregations of proteins in the hippocampus, aberrant structural changes and neuronal connections in synapses, and neuronal degeneration. *Trpm2*^−/−^ mice exhibit learning and memory impairment, enhanced neuronal intrinsic excitability, and imbalanced synaptic transmission. These results respond to long-unanswered questions regarding the potential role of the enzymatic function of TRPM2 in the brain, whose dysfunction evokes protein aggregation. In addition, the present finding answers to the conflicting reports such as neuroprotective or neurodegenerative phenotypes observed in *Trpm2*^−/−^ mice.

**Electronic supplementary material:**

The online version of this article (10.1007/s12035-018-1309-0) contains supplementary material, which is available to authorized users.

## Introduction

Transient receptor potential ion channel subtype M2 (TRPM2, formerly named LTRPC2 or TRPC7) is a multifunctional, nonselective, Ca^2+^ permeable, cation channel [1, 2]. TRPM2 is ubiquitous in the brain, where it is involved in neurite growth during fetal neurodevelopment and functions as an oxidative sensor in neurons [3–5]. Moreover, its genetic or pathogenic dysfunction is associated with bipolar disorder and neurodegenerative diseases, including Parkinson’s and Alzheimer’s [6–9]. TRPM2 is activated by ADP-ribose (ADPR), nicotinamide adenine dinucleotide (NAD^+^), mild heat, or H_2_O_2_ [1, 2, 10]. Being a Ca^2+^-permeable cation channel, when activated by reactive oxygen species (ROS), TRPM2 induces ROS-mediated neuronal degeneration and chemokine production in the macrophages [11, 12]. Thus, genetic ablation of trpm2 leads to neuroprotective effects in β-amyloid- or ischemia-induced brain [7, 13, 14]. On the contrary, some reports show neurodegenerative phenotypes in *Trpm2*^−/−^ mice [8, 15]. However, the reason for the conflicting results is not known.

Unlike other TRP channels, only TRPM2 possesses a pore region and an enzymatic domain, NudT9-H, in its C-terminus region. The NudT9-H region shares significant homology with NudT9 ADPR pyrophosphatase (NudT9) that hydrolyzes ADPR into adenosine monophosphate (AMP) and a ribose [1, 2, 16]. Thus, it is often referred to as a *chanzyme*. Indeed, TRPM2 can hydrolyze ADPR weakly in vitro [1]. However, it has still been veiled on in vivo hydrolase activity of TRPM2 in the brain.

ADPR is a metabolic product of pyridine nucleotides NAD (H) or NADP (H) and reacts readily with various proteins due to the nature of a reactive nucleotide-sugar, inducing protein aggregation [17, 18]. Therefore, its degradation is essential for normal cellular functions [19, 20]. Cellular ADPRs are catabolized by an ADPR pyrophosphatase, a member of the Nudix gene family [19]. Nudix hydrolases remove the deleterious metabolite, ADPR, from biochemical pathways, preventing the excessive accumulation of ADPR in cells [19, 20]. If allowed to accumulate, ADPR tends to react with the lysine and arginine residues of proteins due to the activity of ADP-ribosyltransferase or non-enzymatic way, leading to an excessive mass of protein aggregates [17, 18, 21]. Protein oxidation by oxidative stress in aging and neurodegenerative brains induces oxidized and cross-linked proteins, which further accelerates the formation of protein aggregates [22, 23]. The damaged proteins and lipids are rapidly recycled by the autophagy pathway. Autophagy is a lysosomal proteolytic pathway that is widely involved in the degradation of damaged or defective cellular proteins and organelles [24]. However, the weak proteolytic efficiency of autophagy during aging is now known to induce the accumulation of intracellular waste products, which make individuals susceptible to age-related neurodegenerative diseases, such as Alzheimer’s and Parkinson’s [25]. Thus, ADPR accumulation along with weak autophagic activity would lead to deleterious protein aggregations.

In mammals, ADPR is hydrolyzed by NudT-5 and NudT-9 in the major organs, but not in the brain [19, 20]. As a result, the enzyme responsible for ADPR degradation in the brain has come under question. Given the abundance of TRPM2 in the brain, it is possible that TRPM2 may hydrolyze the reactive ADPR. The present study was thus undertaken to determine the role of TRPM2 in hydrolyzing ADPR in the brain and the possible pathology when this function is chronically impaired.

## Materials and Methods

### Animals

Animal care and handling were carried out according to guidelines issued by the Institutional Animal Care and Use Committee at the Korea Advanced Institute of Science and Technology (KAIST) and Seoul National University.

The generation of mice lacking in TRPM2 for the study is described in the previous report [6]. TRPM2 heterozygous knockout (*Trpm2*^*±*^ ) mice were backcrossed into the C57BL/6J inbred background over 10 generations. Male TRPM2 wild-type (WT, *Trpm2*^*+/+*^) and TRPM2-deficient (*Trpm2*^*−/−*^) mice with a C57BL/6J background were used for the analysis. All of the experiments were performed on 8- to 12-week-old mice unless otherwise indicated. The animals were provided with free access to food and water under a 12:12-h light:dark cycle.

### Extracellular Recordings from Brain Slices

Recordings were obtained of prepared hippocampal (300–330 μm-thick), as described in our previous report [26]. Brain slices were prepared in oxygenated (95% O_2_, 5% CO_2_), cold, artificial cerebrospinal fluid (in mM, 124 of NaCl, 3.5 of KCl, 1.25 of NaH_2_PO_4_, 2.5 of CaCl_2_, 1.3 of MgSO_4_, 26 of NaHCO_3_, and 10 of glucose, at pH 7.4). After allowing 1 h for recovery, the slices were incubated in artificial cerebrospinal fluid, and whole-cell recordings were obtained for the hippocampal CA1 neurons at 32 °C using glass pipette electrodes (3–5 MΩ). To measure the action potentials and miniature excitatory postsynaptic currents (mEPSCs), glass pipettes were filled with a solution, containing (in mM) 135 of K-gluconate, 5 of KCl, 10 of HEPES, 2 of MgCl_2_, 0.3 of Na-GTP, 5 of Mg-ATP, 0.5 of CaCl_2_, and 5 of EGTA (pH 7.3). APs were triggered by injecting currents, ranging from – 150 to + 150 pA, in 30 pA steps using the current clamp mode. The number of spikes that were evoked by the currents injected in the hippocampal neurons isolated from WT or *Trpm2*^−/−^ mice was compared. The mEPSC experiment was performed in the presence of 1 μM of tetrodotoxin, 100 μM of picrotoxin (a GABA_A_ receptor antagonist), and 5 μM of CGP 55845 (a GABA_B_ receptor antagonist). For miniature inhibitory postsynaptic current (mIPSC) measurements, the internal pipette solution contained (in mM) 140 of KCl, 0.5 of CaCl_2_, 2 of MgCl_2_, 5.8 of EGTA, 10 of HEPES, 5 of Mg-ATP, and 0.3 of Na-GTP (pH 7.3). The mIPSC experiment was performed in the presence of 10 μM of DNQX (an AMPA receptor blocker) and 50 μM of D-AP5 (an NMDA receptor antagonist) in the voltage clamp mode. For mEPSC and mIPSC measurements, neurons were voltage-clamped at − 60 mV. Patch-clamp recordings were also performed using a Multiclamp 700B amplifier. Data were filtered at 1 kHz and sampled at 10 kHz using a Digidata 1440A (Axon Instruments). The acquired data were analyzed using a pCLAMP version 10.2 (Axon Instruments) and the Mini Analysis Program (Synaptosoft). For the LTP experiment, field excitatory postsynaptic potentials (fEPSPs) were recorded at hippocampal CA3-CA1 synapses. A bipolar stimulating electrode was placed in the stratum radiatum in the CA1 region, and extracellular field potentials were also recorded in the stratum radiatum using a glass microelectrode (borosilicate glass, 3–5 MΩ, filled with 3 M of NaCl). A baseline stimulation was delivered at an intensity eliciting 40% of the maximum evoked response. A theta-burst LTP was induced by the theta-burst stimulation protocol, consisting of four trains with 10-s intervals between each. Each of the trains was comprised of 10 bursts separated by 200 ms. Each burst included four pulses delivered at 100 Hz.

### Ultrastructural Analysis

The WT and *Trpm2*^*−/−*^ mice (8–12 weeks) were deeply anesthetized with 15% chloral hydrate. They were transcardially perfused with 100 ml of normal saline, followed by 500 ml of a freshly prepared fixative that contained 2% paraformaldehyde and 2.5% glutaraldehyde in a 0.1 M phosphate buffer (pH 7.4). The brains were removed and sectioned with a Vibratome at 500 μm. The sections, including the hippocampus sections, were post-fixed in the same fixative for 3 h, washed in phosphate buffer containing 4.5% sucrose for 15 min (3 × 5 min), and then post-fixed in a 1% OsO_4_ in phosphate buffer for 1 h. Each section was then rewashed in a phosphate buffer containing 4.5% sucrose and dehydrated in a graded alcohol series. During dehydration, the sections were stained en bloc with 1% uranyl acetate in 70% alcohol for 1 h, transferred to propylene oxide, flat-embedded in Epon 812, and cured at 60 °C for 3 days. Small pieces containing the CA1 pyramidal cell layer and stratum radiatum were then cut out and attached to an Epon support for further ultrathin sectioning (Reichert-Jung, Nuβloch, Germany). Ultrathin sections (70–90 nm thick) were collected on 1-hole grids coated with Formvar and examined under an electron microscope (Jeol 1200EX, Tokyo, Japan). Randomly selected neuropil areas, within 70–100 μm from the cell bodies, were photomicrographed at a 40,000× and used for quantification. Three electron micrographs representing 159.9 μm^2^ neuropil regions were taken per mouse. Digital images were captured with a CCD camera (SC1000 Orius; Gatan Inc., Pleasanton, CA) and saved as TIFF files. Image brightness and contrast were adjusted using Adobe Photoshop (Adobe Systems, San Jose, CA).

### Immunohistochemistry

After being anesthetized, the WT and *Trpm2*^*−/−*^ mice (8–12 weeks) were perfused transcardially with saline, followed by 4% paraformaldehyde. Their brains were excised and placed in 4% paraformaldehyde for 24 h. Fixed tissues were then embedded in an OCT compound (Sakura Finetechnical Company, Chuo-Ku, Japan) and placed on slides. Immunohistochemical staining was performed using antibodies to NeuN (Millipore). The brain tissues were first incubated for 1 h in a solution containing 4% bovine serum albumin and 0.05% Tween 20, and then incubated overnight at 4 °C in a solution containing primary antibodies. Finally, sections were incubated with Alexa Fluor 488-conjugated donkey anti-mouse IgG (Invitrogen) and Hoechst 33342 (Invitrogen) for 1 h at room temperature to perform nuclear staining.

### Behavioral Tasks

All behavioral procedures were video-recorded and an experimenter who was unaware of the genotypes analyzed the recorded data.

#### Classical Fear Conditioning Test

As previously described [27], the mice were first habituated in a fear-conditioning apparatus chamber for 5 min and then subjected to a 28-s acoustic conditioned stimulus (CS). A 0.7-mA shock (unconditioned stimulus) was applied to the floor grid for 2 s immediately afterward (Panlab, S.L.U.). Conditioned stimulus-unconditioned stimulus coupling was carried out three times at 60-s intervals. To assess contextual memory, the animals were placed back into the training context 24 h after they received their training. The duration of their fear response (freezing behavior) was measured for 4 min. To assess the cued memory, the animals were placed in a different context (a novel chamber) 24 h after training and their behavior was monitored for 5 min. During the last 3 min of this test, the animals were exposed to the CS. The duration of their freezing behavior was measured throughout the 3-min test. To evaluate the foot-shock intensity, naive animals were placed into the fear-conditioning apparatus chamber and subjected to a series of foot shocks lasting 1 s. The foot shocks gradually increased in amperage (intensity) (0.02, 0.06, 0.1, 0.2, 0.3, 0.4, 0.5, 0.6, 0.7, 0.8, 0.9, and 1.0 mA) at 30-s intervals. The shock intensities that evoked initial sensation responses (flinching and abrupt walking), running, vocalization, and jumping were recorded for each mouse.

#### Y-Maze Test

The Y-maze apparatus consisted of three identical arms. Each arm was 25-cm long, 5-cm wide, and 14-cm high. One of the arms of the maze was briefly closed, and the mice were placed randomly into one of the other arms (start arm) and allowed to explore the maze for 10 min (training session). After 1 h, the mice were replaced in the start arm and allowed to freely explore all three arms for 5 min (test session). The retention times (duration) in each arm were used to assess the spatial memory of the mice. The results are presented as ratios of the amount of time spent in each arm, over the total time spent in all three arms.

#### Novel Object Recognition Memory Test

As previously described [27], the mice were individually habituated to an open-field box (40 × 40 × 40 cm) for 15 min per day for 3 days. During the training trial, two objects were placed in the box, and the mice were allowed to explore the objects for 10 min. A mouse was considered to be exploring an object when its head was less than 1 in. away from the object and facing it. Twenty-four hours later, the mice were returned to the box with the two objects in the same locations, but with one novel object replacing one of the familiar objects. The mice were then allowed to explore the two objects for 10 min. Preference percentages (defined as the time spent exploring an object expressed as a percentage of the total time spent exploring both objects) were used to assess recognition memory.

### Quantification of Metabolites by Triple Quadrupole Mass Spectrophotometer

As previously described, tissue extracts were analyzed by LC-MS/MS [28]. To determine the concentrations of ADPR and AMP in the tissue extracts, hippocampi from WT and *Trpm2*^*−/−*^ mice were briefly treated with 10% (*v*/*v*) trifluoroacetic acid and sonicated. These tissue extracts were centrifuged at 13000 rpm for 20 min, and the supernatants were diluted for analysis. The diluted supernatants were separated using a BEH Amide column (Waters ACQUITY UPLC BEH Amide, 130 Å, 1.7 μm, 2.1 mm × 50 mm). All chromatographic separations were performed at a flow rate of 0.2 mL/min. The column was equilibrated with a 100% buffer B (99.9% acetonitrile/0.1% formic acid), and the tissue extractor was eluted in a 5-min gradient to a 100% buffer A (10 mM of ammonium formate in water). The column was then rinsed with an ammonium formate buffer and re-equilibrated with the acetonitrile/formic acid buffer before the next injection. The following optimal conditions were used for the MS analysis of ADPR, NAD, and AMP: cone gas 150 L/h, nebulizer 7 Bar, and desolvation temperature at 350 °C. The ion transitions used for quantification were m/z 558.17 → 346.01 for ADPR, 662.27 → 540.14 for NAD, and 348.09 → 136.06 for AMP.

### ADPR Hydrolase Activity

The hippocampus tissues from WT and *Trpm2*^*−/−*^ KO mice were suspended in a 1 ml ice-cold lysis buffer, containing 30 mM Tris-HCl (pH 7.0), 150 mM NaCl, and a protease inhibitor cocktail. The suspensions were sonicated for 30 s and centrifuged at 12,000*g* for 10 min at 4 °C. The supernatants were harvested and the pellets were resuspended in the same buffer. The sonication and centrifugation of the insoluble pellets were repeated five times. The supernatants and finally re-suspended pellets were all ultra-centrifugated at 100,000*g* for 1 h at 4 °C. The supernatant was discarded, and the insoluble pellets were twice washed and solubilized by the addition of a lysis buffer, including 1% Triton X-100. Following solubilization, the samples were centrifuged at 120,000*g* for 1 h to obtain soluble fractions. The Nudix activity of lysate from each hippocampus was determined by measuring the conversion of substrate ADPR to AMP using LC-MS/MS. The reaction mixture (260 ul) consisted of 50 mM of Tris-HCl (pH 6.8), 16 mM of MgCl_2_, 40 μM of ADPR, and 40 μg of tissue lysate. After incubation for 1 h at 37 °C, the reaction was stopped by adding EDTA. The protein was removed by Vivaspin (3000 MWCO, Sartorius, Goettingen, Germany) and the product, AMP, was measured, as described in the Methods section above. The ms/ms response of the detected total AMP was removed with those of the sample blank and contaminated with AMP at the standard ADPR.

### Western Blot

The protein lysates from the hippocampi of WT or *Trpm2*^−/−^ mice were prepared in a RIPA cell lysis buffer (GenDEPOT), containing a protease inhibitor cocktail (Roche). These lysates were then subjected to an 8% SDS-PAGE gel. The proteins were transferred to PVDF membranes and then treated for 1 h with a TBS-T solution (20 mM of Tris/HCl, 500 mM of NaCl, 0.1% Tween 20), containing 2–5% skimmed milk powder. They were incubated with primary antibodies against β-actin (Sigma), α-tubulin (Millipore), GAPDH (Santa Cruz), calnexin (Santa Cruz), EEA-1 (Abcam), LAMP-1 (Santa Cruz), or autophagy-related proteins (LC3B:#2755, mTOR: #2972, phospho-mTOR: #5536, Raptor:#2280, AMPKα: #2532, phospho-AMPKα: #2531, AMPKβ1: #12063, phospho-AMPKβ1: #4181, ULK1: #8054, phospho-ULK1 (Ser555): #5869, phospho-ULK1 (Ser757): #14202, from Cell Signaling) at 4 °C on a rotary shaker overnight. The membranes were washed three times in a TBS-T solution, incubated with a secondary antibody for 1 h, and then treated with WEST-ZOL® ECL solution (iNtRON biotech). Blots were analyzed using an ImageQuant™ LAS 4000 chemiluminescence (GE Healthcare).

### Experimental Design and Statistical Analysis

All of the results are shown as means ± SEMs. The unpaired two-tailed Student’s *t* test was used to determine the statistical differences between the two means. A one-way or two-way analysis of variance (ANOVA) with Tukey’s post hoc analysis was used to conduct multiple comparisons of the means. Mann-Whitney or Kruskal-Wallis analysis was used to conduct multiple comparisons of the means with small number of experiments (*n*≤ 6). Statistical significance was accepted for *p* value of < 0.05.

## Results

### Reduced Catabolism of ADPR in the *Trpm2*^−/−^ Mice Brains

**Fig. 1:**
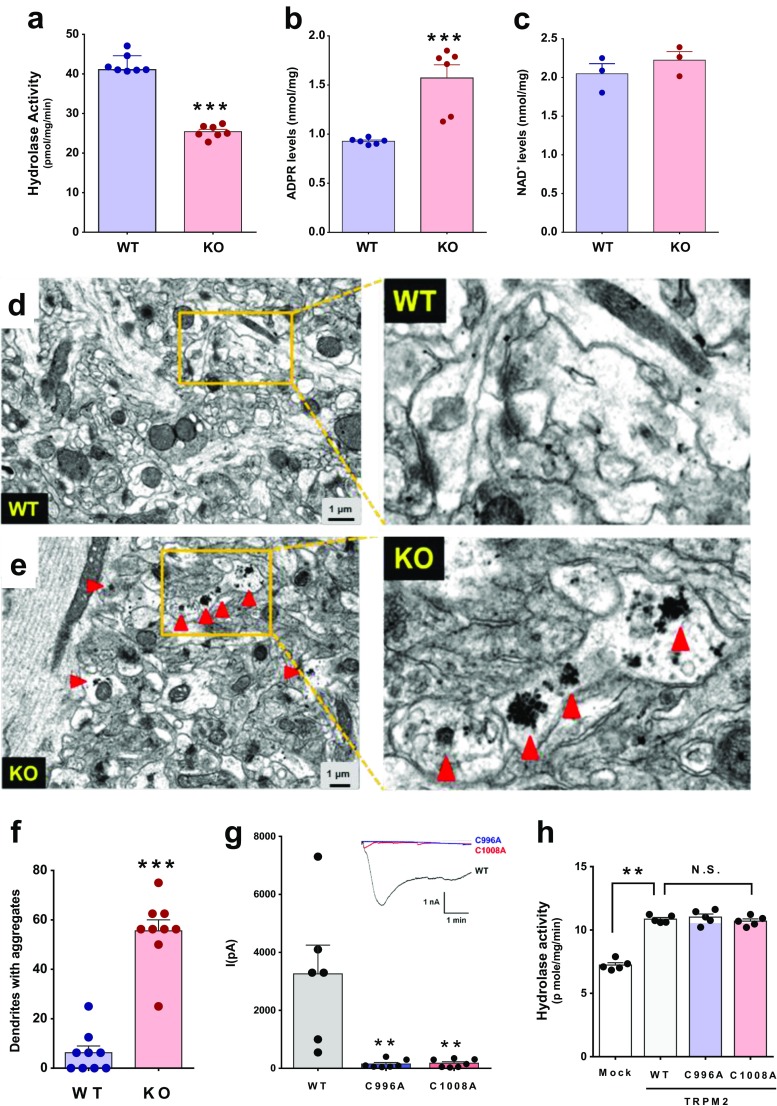
Reduced ADPR-hydrolyzing activity and accumulated proteins in the hippocampi of *Trpm2*^*−/−*^ mice. **a** ADPR hydrolase activities in the brains of WT (*n* = 7) and *Trpm2*^*−/−*^ mice (*n* = 7). Hippocampus lysates (40 μg) were treated with ADPR (40 μM). Levels of AMP (an immediate metabolic product of ADPR hydrolysis) were used as a surrogate of hydrolase activities. ***p < 0.001, Student’s *t* test. **b** ADPR levels in the hippocampi of WT (*n* = 5) and *Trpm2*^*−/−*^ mice (*n* = 5). ***p < 0.001, Student’s *t* test. **c** Hippocampal levels of NAD^+^ (an upstream substrate of ADPR) in WT (*n* = 5) and *Trpm2*^*−/−*^ mice (*n* = 5). **d**, **e** Brain slices taken from the hippocampal CA1 regions (stratum radiatum) of 8~12-week-old WT (**d**) and *Trpm2*^*−/−*^ mice (**e**) were subjected to ultrastructural electron microgram analysis. The general appearance of the neuropils was similar for both genotypes. However, prominent inclusion bodies (arrowheads) were observed throughout the dendrites of the hippocampal neurons of *Trpm2*^*−/−*^ mice. Right panels, boxed areas are shown at a higher magnification. Note that spherical protein aggregates of variable sizes were observed in dendrites in the hippocampal neurons of *Trpm2*^*−/−*^ (KO) mouse brain. **f** Summary of the inclusion body occurrence in the hippocampal neurons of WT and KO mice. ***p < 0.001, Student’s *t* test. **g** ADPR-induced currents of HEK-293T cells transfected with WT, C996A, or C1008A *Trpm2*. Inset, representative current traces of each group. **p < 0.01, Kruskal-Wallis test. **h** ADPR-hydrolase activities in the lysates of WT, C996A, or C1008A *Trpm2* overexpressing HEK-293 T cells. **p < 0.01, Kruskal-Wallis test

To determine the capacity of the brain to hydrolyze ADPR, we added ADPR to the lysates of hippocampal cells isolated from WT and *Trpm2*^−/−^ mice and measured the conversion of substrate ADPR to AMP, an immediate metabolic product of ADPR hydrolysis. As shown in Fig. 1a, the ADPR hydrolyzing activity was weaker in the brains of the *Trpm2*^−/−^ mice than in the brains of the WT mice. This is because the ADPR turnover to AMP in the *Trpm2*^−/−^ mice brains was significantly lower (*p* < 0.001, Student’s *t* test). Consistent with this, a marked accumulation of ADPR (*p* < 0.001, Student’s *t* test) was observed in the brains of the *Trpm2*^−/−^ mice (Fig. 1b). However, the levels of NAD^+^, a metabolic precursor of ADPR, were similar for the two genotypes (Fig. 1c). Therefore, TRPM2 appears to engage in the hydrolase activity of ADPR in the brain, the disruption of which results in increased levels of ADPR in the brain.

### Accumulated Aggregates in *Trpm2*^−/−^ Mice Brains

Because ADPR reacts with various proteins due to the nature of a reactive nucleotide sugar [17, 18], we assumed that the accumulation of ADPR might induce protein aggregation in Trpm2^−/−^ mice brains. To confirm the possibility, we conducted ultrastructural analysis using electron microscopy. Strikingly, we observed the presence of multiple inclusion bodies in the dendrites of the hippocampal neurons in the brains of young adults (8~12 week old) (Fig. 1d–e) [29]. These round and varied protein aggregates were observed throughout dendrites, including the spines. The protein aggregates appeared different to the neurofilamentary tangles of Tau [30]. The proportion of hippocampal neurons containing inclusion bodies was about 9 times higher in *Trpm2*^−/−^ mice than in the hippocampal neurons of WT mice (Fig. 1f). In every 1000 μm^2^, 55.6 ± 4.8 (*n* = 9) dendrites in the hippocampus of the *Trpm2*^−/−^ mice showed inclusion bodies, whereas for WT mice, only 6.3 ± 2.9 (*n* = 9) dendrites in the hippocampus contained such aggregates (*p* < 0.001, Student’s *t* test).

### ADPR Hydrolyzing Activity Is Not Associated with its Channel Activity

We also examined whether the TRPM2 channel activity is functionally associated with the enzymatic activity of TRPM2. To determine this, we mutated Trpm2 at the pore region to block channel activity and then tested whether these mutants retain the ADPR hydrolyzing activity. We constructed two TRPM2 mutants that had mutations at C996 or C1008 in the pore region as previously reported [31]. The application of 100 μM ADPR in the pipette solution robustly induced the currents of wild-type (WT) *Trpm2,* but not the two mutants, C996A and C1008A (Fig. 1g). As shown in Fig. 1h, the lysate isolated from WT *Trpm2* overexpressing HEK293T cells showed significantly higher ADPR hydrolysis activity than that of mock-transfected cells (*p* < 0.01, Kruskal-Wallis test). More importantly, the hydrolysis activities of C996A and C1008A mutants of *Trpm2* were not different from that of the WT *Trpm2* overexpressing cells. Therefore, these data suggest that channel activity appears to be independent of the ADPR-hydrolysis activity of TRPM2.

### Neuronal Loss in the Hippocampus of *Trpm2*^−/−^ Mice

**Fig. 2:**
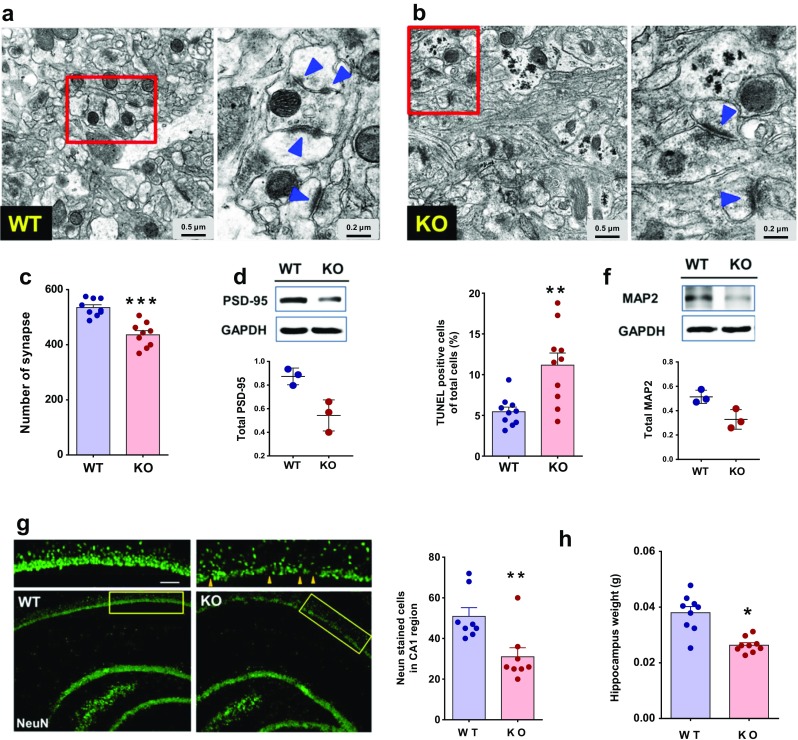
Neurodegeneratoin in the hippocampi of *Trpm2*^*−/−*^ mice. **a**, **b** Shape of individual synapses of both WT (**a**) and KO (**b**) mice. The overall shapes of individual synapse of both genotypes are similar. However, small excitatory synapses with short post-synaptic density (< 0.2 μm in length, arrowheads) are less frequently observed in WT than in KO mice. **c** Summaries of the lengths of synapses in hippocampal brain slices from WT and KO mice. ***p < 0.001, Student’s *t* test. **d** Protein levels of PSD-95, a key component composed of post-synaptic density, in WT and KO mouse brains. The Western blot showed a reduced expression of PSD-95 in KO mouse brains. Bars represent the mean +− S.D. **e** Number of TUNEL positive neurons in the hippocampi of both genotypes. **p < 0.01, Student’s *t* test. **f** Protein levels of microtubule-associated protein 2 (MAP 2), a neuron specific cytoskeletal protein in WT and KO mouse brains. The protein showed a reduced expression in KO mice brains. **g** Immunoreactivity of NeuN antibody (green) in the hippocampal neurons of WT and KO mice. Right panel, comparison of the number of NeuN-positive neurons in every 10,000 μm^2^ in CA1 regions of both genotypes. **p < 0.01. Scale bar represents 50 μm. **h** Wet weights of hippocampi isolated from both genotypes. *p < 0.05, Student’s *t* test

Because of aggregates in the hippocampi of the *Trpm2*^−/−^ mice, we expected structural changes in the hippocampal neurons. As expected, structural changes were also found in the hippocampal synapses of the *Trpm2*^−/−^ mice (Fig. 2b), compared to the hippocampi of WT mice (Fig. 2a). The number of synapses declined in the hippocampi of the *Trpm2*^−/−^ mice compared to the WT mice (*p* < 0.001, Student’s *t* test) (Fig. 2c). Furthermore, the levels of cytoskeletal proteins, known for composing dendritic spines, such as postsynaptic density 95 (PSD-95), were also lower in the hippocampi of the *Trpm2*^−/−^ mice (Fig. 2d). Neuronal degeneration was prevalent in the hippocampi of the *Trpm2*^−/−^ mice, as there was a marked increase in the TUNEL stain found in the *Trpm2*^−/−^ mice compared to the WT mice (Fig. 2e). In the hippocampi of *Trpm2*^−/−^ mice, the loss of hippocampal neurons was evident because of the reduced expression of the microtubule-associated protein 2 (MAP 2), a neuron-specific cytoskeletal protein enriched in dendrites (Fig. 2f). Similarly, when stained with NeuN, a marker for neurons, the number of neurons in the CA1 region of the hippocampus was less in the *Trpm2*^−/−^ mice brains, further supporting the loss of hippocampal neurons in *Trpm2*^−/−^ mice (Fig. 2g). As expected with neuronal loss, the weight of the hippocampi of the *Trpm2*^−/−^ mice was significantly less than that of the WT mice (*p* < 0.05, Student’s *t* test) (Fig. 2h). These results thus suggest that neurons undergo degeneration, along with structural changes, in the dendritic spines of the hippocampi of *Trpm2*^−/−^ mice.

### Altered Excitability and Synaptic Transmission in *Trpm2*^−/−^ Mice

**Fig. 3:**
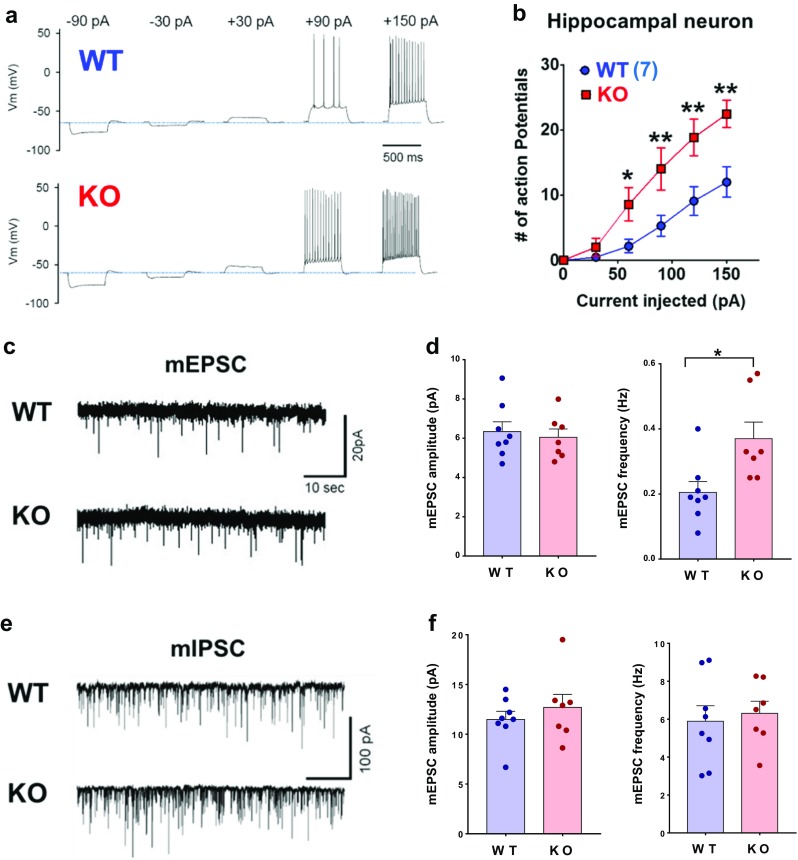
*Trpm2* disruption alters neuronal excitability and synaptic transmission. **a** Traces of the membrane potentials of hippocampal neurons isolated from the brains of WT and KO mice. **b** Depolarizing currents were injected into neurons after forming whole cells. Summary of the number of action potentials in hippocampal neurons of both genotypes generated by the depolarizing current injections. *p < 0.05, **p < 0.01. **c** Sample traces of mEPSCs in CA1 pyramidal neurons in brain slices from WT and KO mice. **d** Summaries of the amplitudes (*left*) and frequencies (*right*) of mEPSCs from WT (*n* = 8) and KO (*n* = 7) mice. *p < 0.05, Student’s *t* test. **e** Sample traces of mIPSCs from WT and KO mice. **f** Amplitudes (*left*) and frequencies (*right*) of mIPSCs in CA1 pyramidal neurons from WT (*n* = 8) and KO (*n* = 7) mice

Because structural abnormality develops alterations in neuronal excitability or synaptic activity [32], changes in the neuronal excitability and synaptic transmission were determined in the hippocampi of 7- to 8-week-old WT and *Trpm2*^*−/−*^ mice. The number of action potentials induced by injecting currents of 150 pA (30 pA increment) were significantly greater in the hippocampal CA1 neurons of the *Trpm2*^−/−^ mice (*F*_(1,15)_ = 27.6, *p* < 0.001, two-way ANOVA, *n* = 7) than in those of the WT mice (*n* = 10) (Fig. 3a–b). Synaptic activity in the *Trpm2*^−/−^ mice was examined after recording mEPSCs and mIPSCs in the hippocampal CA1 neurons. The frequencies of mEPSCs were significantly higher in the hippocampal CA1 of *Trpm2*^−/−^ mice (*p* < 0.05, *n* = 7) than in those of WT mice (*n* = 8). In contrast, the mEPSC amplitudes were unaffected (Fig. 3c–d). The amplitudes and frequencies of mIPSCs were not different in the hippocampal CA1 neurons of the *Trpm2*^−/−^ and WT mice (Fig. 3e–f).

### Altered Cognitive Function and Synaptic Plasticity in *Trpm2*^−/−^ Mice

**Fig. 4:**
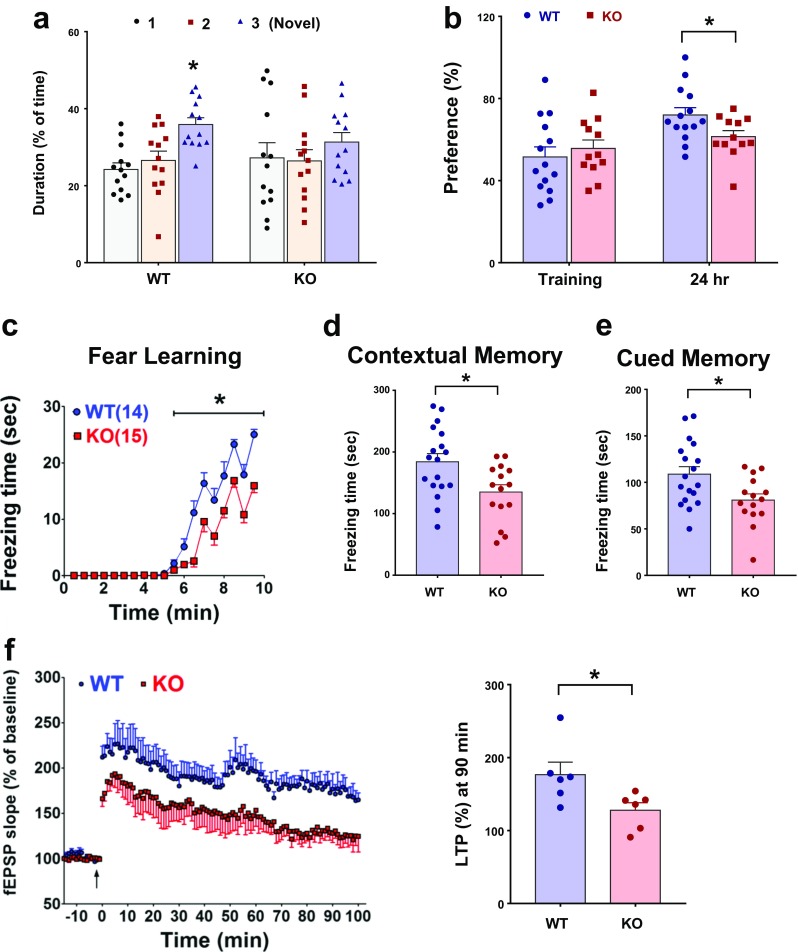
*Trpm2* disruption alters learning and memory. **a** Y-maze test. KO mice showed impaired performance by spending the same amount of time in novel or familiar arms, whereas WT mice spent more time in the novel arm (1, start; 2, known; 3, novel arm). *p < 0.05, *n* = 13. **b** Novel object recognition task. During training, no difference was observed between WT and KO mice in terms of exploratory preference. In contrast, after a 24-h retention period, KO mice (*n* = 12) showed less preference for the novel object than WT mice (*n* = 14). *p < 0.05. **c**–**e** Fear-conditioning test. **c** KO mice (*n* = 15) showed retarded learning on the day of training compared to WT mice (*n* = 14) (*p < 0.05). **d** Contextual fear-conditioning testing 24 h after training. KO mice displayed less freezing behavior than WT mice (*p < 0.05). **e** Cued fear-conditioning test 24 h after training. KO mice displayed less freezing behavior than WT mice during tone presentation (*p < 0.05). **f** LTP elicited by theta burst stimulation. fEPSPs were recorded in hippocampal CA3-CA1 synapses. The potentiation in KO mice (*n* = 8) was significantly less than in WT mice (*n* = 8). *p < 0.05

Because altered synaptic transmission is associated with cognitive impairment [33], we investigated the learning/memory performance and synaptic activity in the hippocampus. The spatial memory, according to the performance in the Y-maze task, was significantly poorer in *Trpm2*^−/−^ mice than in WT mice: the *Trpm2*^−/−^ mice showed no preference for the novel arm (*p* = 0.51, one-way ANOVA, *n* = 13), whereas the WT mice spent more time in the novel arm (*p* < 0.001, one-way ANOVA, *n* = 13) (Fig. 4a). Memory loss in the *Trpm2*^−/−^ mice was also observed in the novel object recognition-memory task, as the mice showed less preference for the novel object than the WT mice 24 h after training (Fig. 4b). In the fear-conditioning test, the *Trpm2*^−/−^ mice (*n* = 15) exhibited impaired acquisition of fear learning on the training day compared to the WT mice (*F*_(1, 27)_ = 75.81, *p* < 0.001, two-way ANOVA, *n* = 14) (Fig. 4c). There was no difference in the foot-shock sensitivity between the two groups (Supplementary Fig. 1). The *Trpm2*^−/−^ mice also showed reduced fear memory when tested with a contextual (Fig. 4d) or a cued (Fig. 4e) memory task 24 h after training. The impaired learning and memory of the *Trpm2*^−/−^ mice suggests the involvement of activity-dependent long-term synaptic transmission, such as long-term potentiation (LTP). As a result, we also examined synaptic function at hippocampal CA3-CA1 synapses in *Trpm2*^−/−^ mice. The *Trpm2*^−/−^ mice displayed a reduced potentiation of theta burst stimulation-induced LTP compared to the WT mice (*Trpm2*^*−/−*^,127.9 ± 10.2% vs WT, 176.6 ± 17.2% of baseline at 90 min, *p* < 0.05, *n* = 8) (Fig. 4f). These results indicate that TRPM2 deletion impairs cognitive function and long-term synaptic transmissions.

### Reduced Autophagy in the *Trpm2*^−/−^ Mouse Brain

**Fig. 5:**
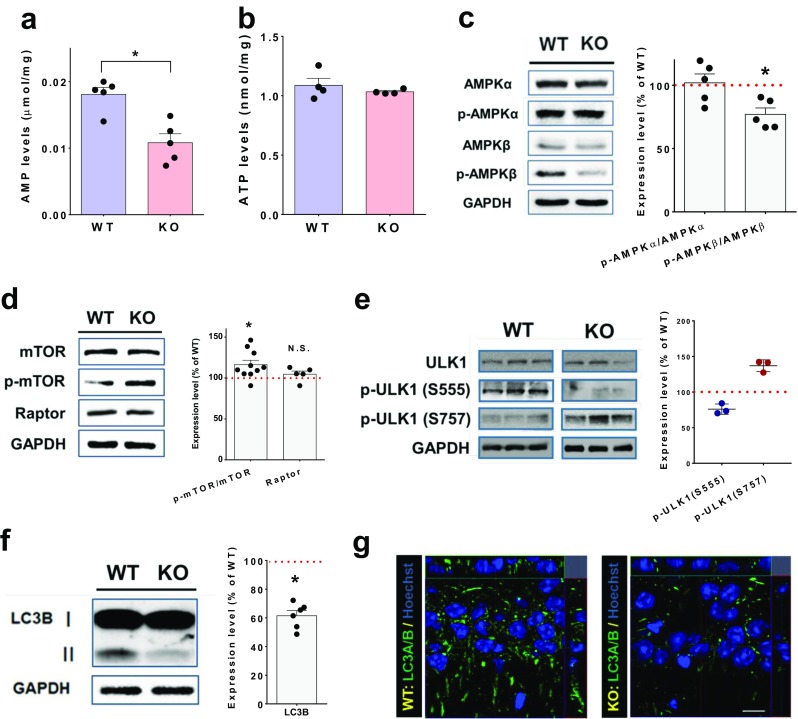
Reduced autophagy activity in the hippocampi of *Trpm2*^*−/−*^ mice. **a** AMP levels in the hippocampi of WT (*n* = 5) and *Trpm2*^*−/−*^ mice (*n* = 5). **p < 0.01, Kruskal-Wallis test. **b** ATP levels in the hippocampi of WT (*n* = 4) and *Trpm2*^*−/−*^ mice (*n* = 4). **c**–**f** Protein levels of key components required for the formation of autophagy in the brains of *Trpm2*^*−/−*^ mice. Western blots of phosphorylated AMPK-α and AMPK-β (**c**), phosphorylated mTOR (**d**), phosphorylated ULK-1 (**e**), and microtubule-associated protein light chain 3B-II (LC3B-II) (**f**). **g** Immunoreactivity against LC3 antibody (green) in the hippocampi of WT and *Trpm2*^*−/−*^ mice

As the level of aggregated proteins was observed in the hippocampi of *Trpm2*^−/−^ mice, we assumed that the protein aggregation might closely be associated with the autophagy pathway that is widely involved in the degradation of defective cellular proteins or organelles [24]. Because the level of AMP is a key substrate signal to induce the autophagy pathway [34, 35], the levels of AMP and ATP in wild-type and *Trpm2*^−/−^ mice brains were measured. A significant reduction (*p* < 0.01, Student’s *t* test) in AMP was observed in the brain of the *Trpm2*^−/−^ mice compared to that of the wild-type mouse brain (*p* < 0.05, Mann-Whitney test) (Fig. 5a). However, there was no difference in the level of ATP in the brains of both genotypes, suggesting that a reduced AMP level is not derived from ATP (Fig. 5b). Because AMP is essential for controlling autophagy via the AMP-activated protein kinase (AMPK)/mTOR pathway [24, 34, 35], we determined the autophagic activity in the brains of both genotypes. The phosphorylated β-form of AMPK was significantly decreased in the *Trpm2*^−/−^ mice (*p* < 0.05, Kruskal-Wallis test) (Fig. 5c). In addition, the level of phosphorylated mTOR, known to be suppressed by AMPK, was significantly increased (*p* < 0.05, Student’s *t* test, *n* = 10) (Fig. 5d). Because AMPK and mTOR coordinate the phosphorylation of ULK1, a key regulator for autophagosome formation [36–38], we compared the phospho-ULK1 on the Ser555 residue (phosphorylated residue by AMPK) and on the Ser757 residue (phosphorylated residue by mTOR) in hippocampal extracts isolated from WT and *Trpm2*^−/−^ mice. As shown in Fig. 5e, the phosphorylation of ULK1 at Ser555 was markedly decreased, whereas phosphorylation of ULK1 on Ser757 was markedly increased in the hippocampus from *Trpm2*^−/−^ mice, which indicates that ULK1 activity is reduced in the hippocampus of *Trpm2*^−/−^ mice.

Because ULK1 is known to regulate the formation of autophagosomes [24, 39], we further checked the level of microtubule-associated protein light chain 3B (LC3B), a key component required for phagophore formation. Indeed, LC3B was significantly reduced in the *Trpm2*^−/−^ mice (*p* < 0.05, Student’s *t* test) (Fig. 5f), whereas the expression levels of markers for endoplasmic reticulum, early endosomes, and lysosomes were unchanged (Supplementary Fig. 2). Moreover, there was no difference in the levels of up-stream signals, including PI3 kinase, Akt, and Erk (Supplementary Fig. 3) [24, 40]. Consistently, the number of autophagosome puncta stained with LC3B antibody was reduced in the sections of the *Trpm2*^−/−^ mice brains (Fig. 5g).

### Reduced ADPR Hydrolase and Autophagy Activities in Hippocampal Cultures

**Fig. 6:**
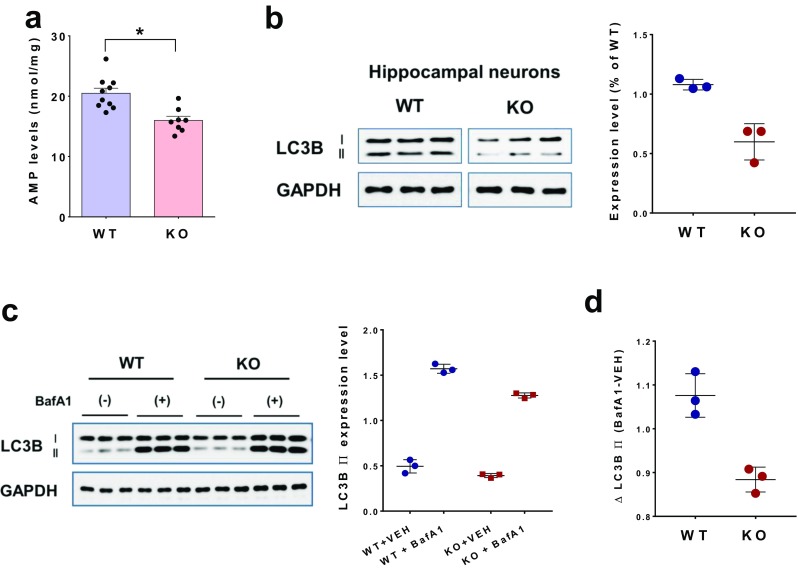
Reduced ADPR catabolism and autophagy in cultured hippocampal neurons isolated from *Trpm2*^*−/−*^ mice. **a** AMP levels in cultured hippocampal neurons of WT (*n* = 10) and *Trpm2*^*−/−*^ mice (*n* = 8). *p < 0.05, Student’s *t* test. **b** Western blots of LC3B-II in cultured hippocampal neurons of WT (*n* = 3) and *Trpm2*^*−/−*^ mice (*n* = 3). The level of LC3B-II was significantly reduced in TRPM2-deficient neurons compared to that of WT. **c** LC3B-II turnover in cultured hippocampal neurons of WT (*n* = 3) and *Trpm2*^*−/−*^ mice (*n* = 3). Western blots (left) and quantitative comparison (right) of the levels of LC3B-II in the presence and absence of bafilomycin (BafA1). **d** The difference in LC3B-II (BafA1 treated—vehicle (VEH) treated) was reduced in the *Trpm2*-deficient hippocampal neurons comparing to WT neurons

We then measured ADPR-hydrolase and autophagy activities in primary cultures of hippocampal neurons isolated from WT and *Trpm2*^−/−^ embryos. Consistent with the results of the in vivo study (Fig. 5a), the level of AMP, a metabolic product of ADPR, was reduced in cultured hippocampal neurons from *Trpm2*^−/−^ mice compared to that from WT mice (*p* < 0.05, Student’s *t* test) (Fig. 6a). The level of LC3B was greatly reduced in *Trpm2*^−/−^ hippocampal neurons compared with that from WT neurons (Fig. 6b). In addition, we also determined the autophagy activity in primary cultures of hippocampal neurons from both genotypes. Autophagy flux (ΔAF) was measured as an indication of autophagy activity that refers to the whole process of autophagy including autophagosome formation, maturation, fusion with lysosome, and degradation [41]. Thus, ΔAF can be detected by LC3B-II turnover in the presence and absence of lysosomal degradation inhibitor, bafilomycin A1. As shown in Fig. 6c, the levels of LC3B-II in both genotypes were increased after bafilomycin A1 treatment. However, ΔAF, the difference between LC3B-II with bafilomycin A1 treatment and that with vehicle treatment was reduced in *Trpm2*^−/−^ hippocampal neurons compared to that of WT neurons, suggesting the decreased autophagosome synthesis in *Trpm2*^−/−^ hippocampal neurons.

## Discussion

The present study demonstrates that TRPM2 functions as a chanzyme that hydrolyzes ADPR in vivo. The genetic disruption of TRPM2 accumulates ADPR, but reduces AMP levels. This induces protein aggregation and lipid accumulation, morphological changes in the spine, and neuronal degeneration, and enhances neuronal excitability and synaptic activity. These pathologic events, in turn, become neural substrates for impaired learning and memory in *Trpm2*^−/−^ mice. Thus, the present study suggests that the enzymatic activity of TRPM2 hydrolyzing ADPR is required to maintain normal brain functions. In addition, the role of TRPM2 as an enzyme to remove neurotoxic ADPR provides the possible explanation for the conflicting phenotypes, neuroprotection, or neurodegeneration, in *Trmp2*^−/−^ mouse brain.

### ADPR Metabolism

ADPR pyrophosphatase is a member of the Nudix superfamily, members of which hydrolyze substrates with a nucleoside diphosphate linked to another moiety “X” (Nudix) [19]. Nudix hydrolases remove the deleterious metabolite, ADPR, from biochemical pathways [19, 20]. Thus, ADPR pyrophosphatases act to prevent excessive ADPR accumulation. If allowed to accumulate, ADPR, a reactive nucleotide-sugar, tends to react with the lysine and arginine residues of proteins by an ADP-ribosyltransferase or a non-enzymatic way [17, 18]. Of the known ADP-ribosylated proteins, globular actin is a known target of ADP-ribosylation in rat brains [42, 43]. The ADP-ribosylation of globular actin hinders actin polymerization, which leads to cytoskeletal disruption and, eventually, cell death [44]. In the present study, aggregated proteins were found in the hippocampal neurons of *Trpm2*^−/−^ mouse brains, which also contained elevated levels of ADPR. Thus, we believe that actin could be one of these aggregated proteins induced by increased ADPR.

### Low Autophagic Formation in *Trpm2*^−/−^ Mice Brains

Autophagy is widely involved in the degradation of damaged or defective cellular proteins and organelles. The reduction in the proteolytic efficiency of autophagy during aging induces the accumulation of intracellular waste products. Thus, low autophagy activity makes individuals more susceptible to age-related neurodegenerative diseases, such as Alzheimer’s or Parkinson’s [25]. The formation of autophagosomes is regulated by ATG, whose expression is suppressed by the mTOR complex [24]. In addition, AMPK that is activated by AMP is an inhibitor of the mTOR complex [24]. In the present study, elevated activity of mTOR, concomitant with low levels of AMPK and AMP, was observed in the brains of *Trpm2*^−/−^ mice. Reduced autophagy formation, as shown by LC3B, was also observed in the brains of *Trpm2*^−/−^ mice. Thus, the reduced ADPR hydrolyzing activity is likely responsible for the reduced autophagic activity in *Trpm2*^−/−^ mice brains. Furthermore, this impaired autophagic formation may account for the protein aggregation found in *Trpm2*^−/−^ brains.

### Protein Accumulation in *Trpm2*^−/−^ Mice Brains

During aging, cellular oxidative stress increases significantly in the brain, which induces protein oxidation [45]. The oxidized proteins are prone to cross-linking with each other and becoming non-functional [45]. The accumulation of aggregated material is observed in the broad regions of the aged brain and is a major risk factor in neurodegeneration [23]. Because TRPM2 is activated by ROS, it is considered to be an oxidative sensor responding to oxidative stress [4]. Consistent with this, the oxidant condition regulates cell survival and death via TRPM2-dependent Ca^2+^ influx [4]. TRPM2 also acts as a chanzyme to remove ADPR, a reactive metabolite induced by oxidative stress. Thus, in the brain, excessive oxidative stress activates TRPM2 and generates AMP in the cytosol, which in turn stimulates the autophagy-related signaling pathway to remove aggregated, oxidized proteins (Supplementary Fig. 4). Therefore, the function of TRPM2 as a sensor and an enzyme is required for the normal functioning of the brain.

### Functional Roles of TRPM2 in Neurodegeneration

There are conflicting reports in describing the role of TRPM2 in neuroprotection or neurotoxicity. Ostapchenko and colleagues showed that TRPM2 deficiency rescues β-amyloid oligomer-mediated neurotoxicity such as synaptic loss and spatial memory deficits in double transgenic APP/PS1 mice [7]. TRPM2 deficiency or pharmacological inhibition attenuates infarct size, neuronal loss, and memory impairment after stroke induced by transient global ischemia [13, 14, 46]. Thus, the TRPM2 loss appears to protect β-amyloid-induced or ischemia-induced neurotoxicity. On the contrary, TRPM2 deficiency leads to impaired synaptic transmission by abnormal regulation of long-term depression [47] and fails to reduce neurological outcomes in mice after permanent middle cerebral artery occlusion [15]. A similar controversy over the role of TRPM2 loss on cell death extends to the heart. TRPM2 deficiency exacerbates mitochondrial dysfunction induced by ischemia-reperfusion [48] whereas TRPM2 deficiency attenuates infarct size in the reperfused myocardium [49]. In the present study, we observed a neuronal and synaptic loss in the hippocampus accompanied by memory loss in *Trpm2*^*−/−*^ mice. The two basic functions of TRPM2 may account for the conflicting results. TRPM2 is activated by ADPR or ROS thus resulting in Ca^2+^ influx, which leads to cell death if prolonged. On the other hand, TRPM2 is an enzyme to remove ADPR, which otherwise leads to cell death because of non-specific ribosylation of structural proteins or disruption in autophagic activity as proposed in this study. Thus, the TRPM2 deletion will result in either cell protection or cell degeneration depending on the two different functions. Experimental conditions such as types of intervention, duration of the treatment, or experimental conditions may lead to different outcomes.

## Electronic supplementary material

ESM 1 (PPTX 1029 kb)
