# Supplementary material for: Trpm2 Ablation Accelerates Protein Aggregation by Impaired ADPR and Autophagic Clearance in the Brain
Source: Mol Neurobiol. 2018 Sep 13;56(5):3819–32. doi: 10.1007/s12035-018-1309-0 (PMC6477016; doi:10.1007/s12035-018-1309-0)
Supplement: Supplementary file 1 — (PPTX 1029 kb) [file 12035_2018_1309_MOESM1_ESM.pptx]

## Slide 1
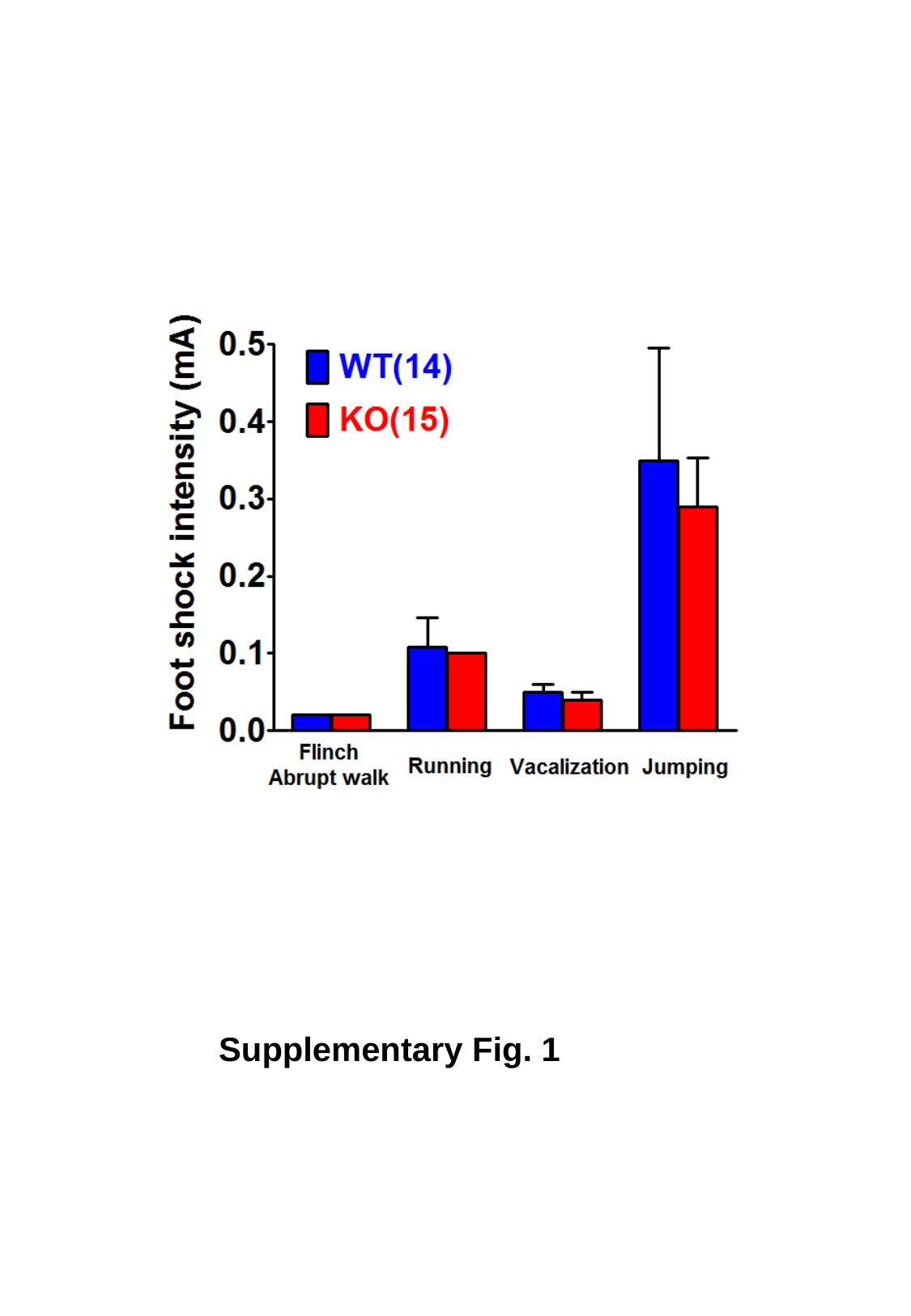

Supplementary Fig. 1

## Slide 2
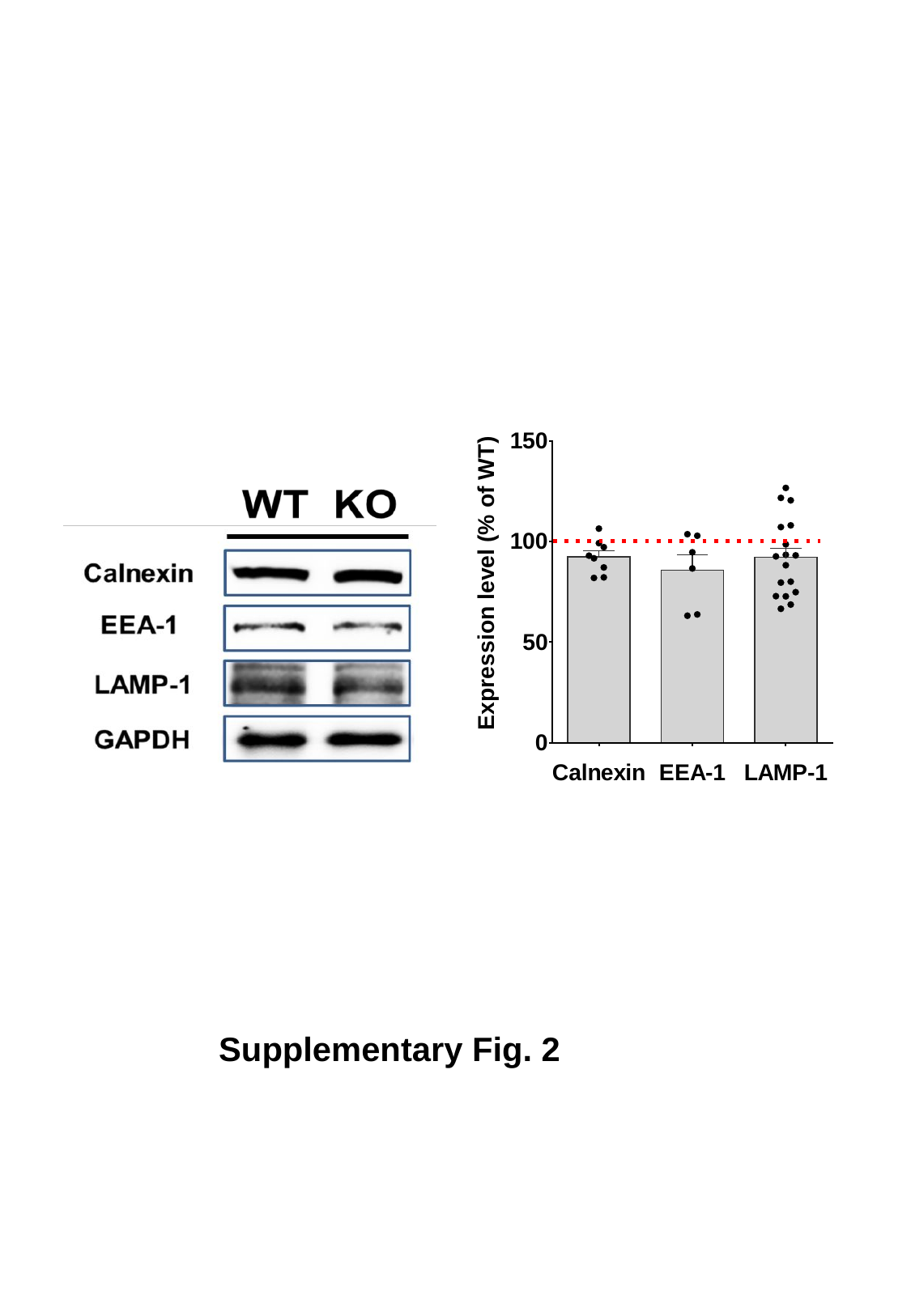

Supplementary Fig. 2

## Slide 3
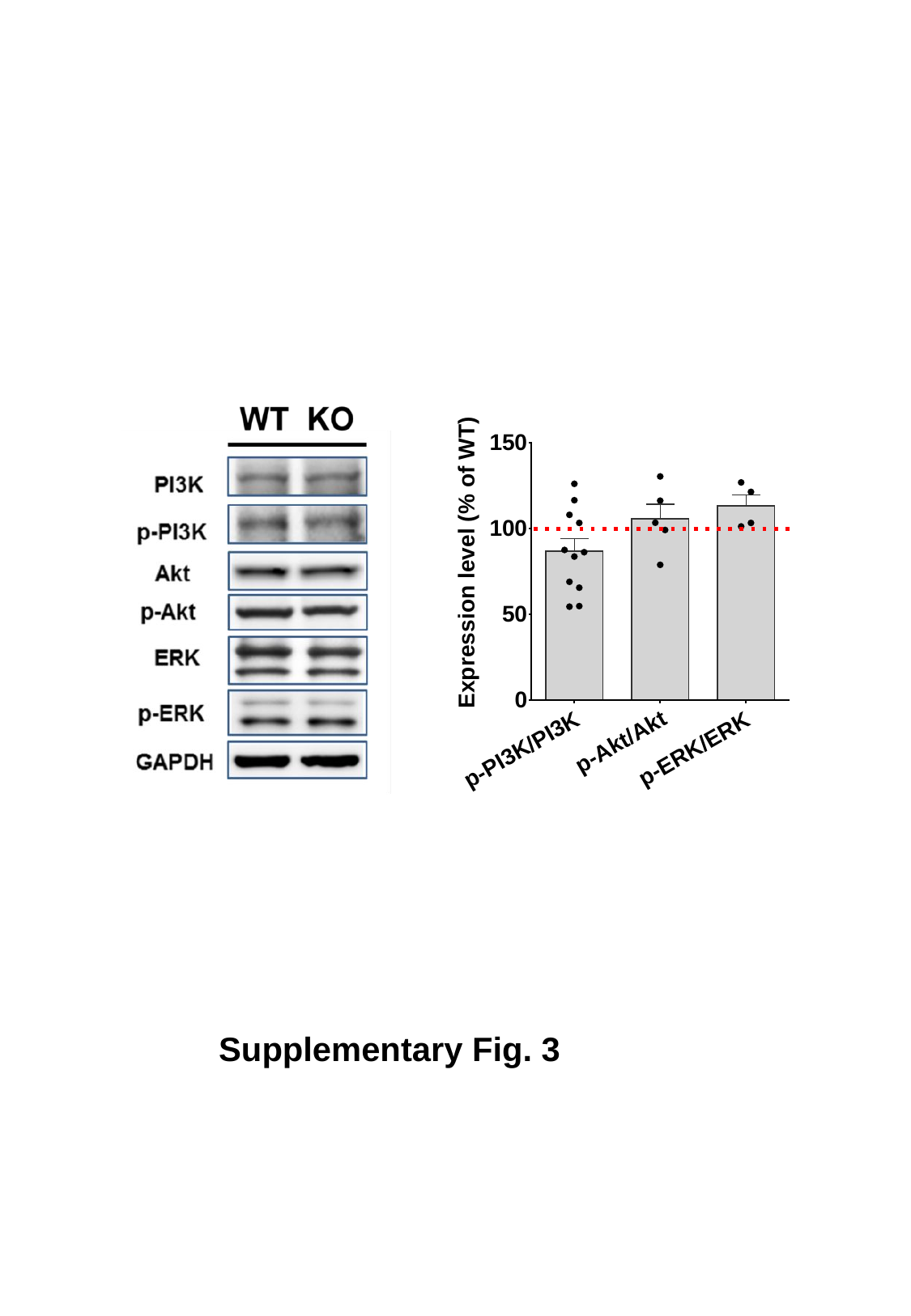

Supplementary Fig. 3

## Slide 4
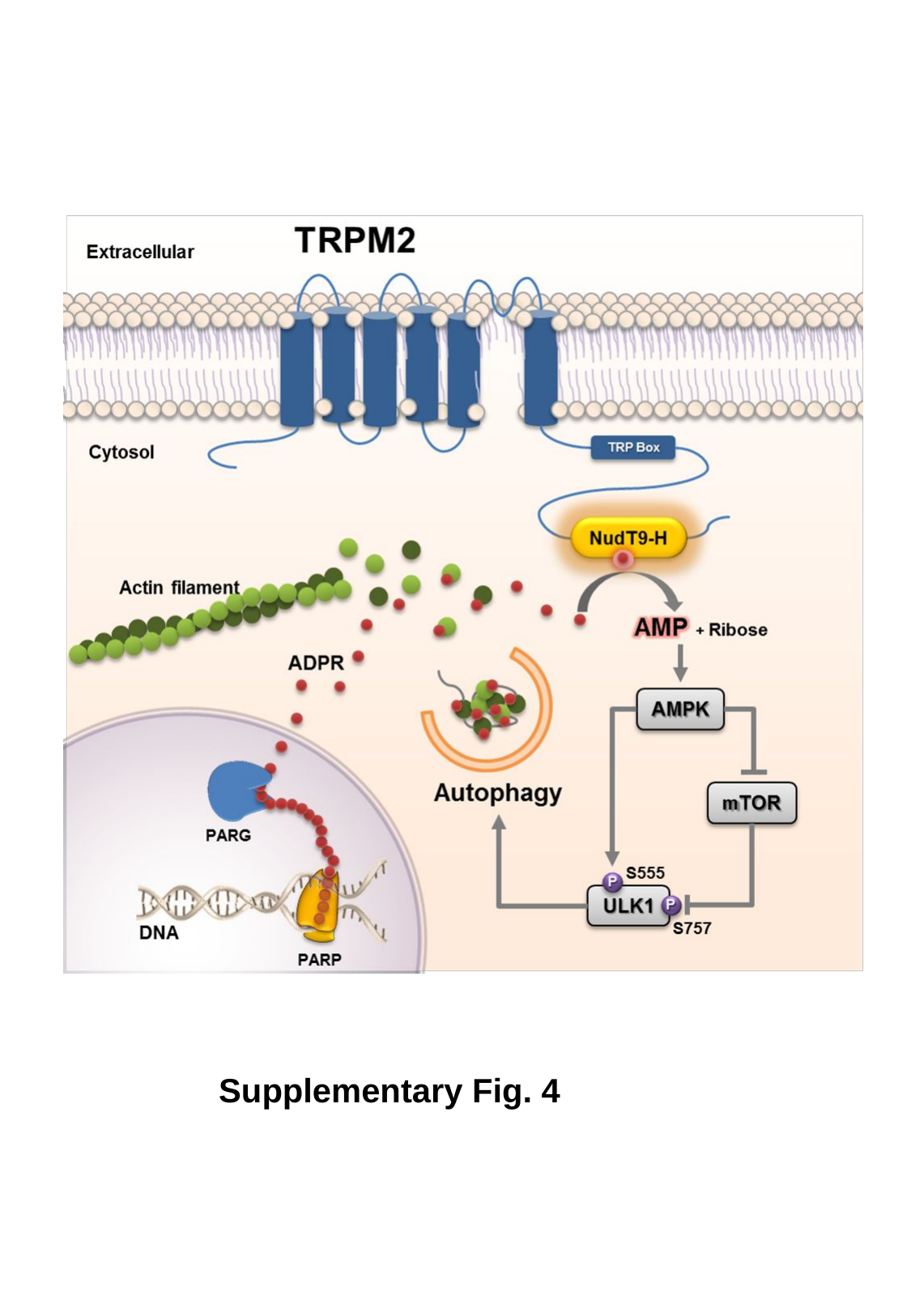

Supplementary Fig. 4
